# Effect of Brassinolide on Soil Microorganisms in Millet Field Polluted by Tribenuron-Methyl

**DOI:** 10.3390/microorganisms11071829

**Published:** 2023-07-18

**Authors:** Xi’e Song, Junli Cao, Shuai Guo, Hao Wang, Qianhui Dong, Pingyi Guo, Xiangyang Yuan

**Affiliations:** 1 College of Agriculture, Shanxi Agricultural University, No. 81 Longcheng Street, Xiaodian District, Taiyuan 030031, China; 2 Shanxi Center for Testing of Functional Agro-Products, Shanxi Agricultural University, No.79, Longcheng Street, Taiyuan 030031, China

**Keywords:** tribenuron-methyl, brassinosteroids, soil microbiome, enzyme activities

## Abstract

Tribenuron-methyl is used to control broad-leaved weeds and has a promising application prospect in millet fields. However, its negative impact on soil ecology cannot be ignored. Brassinosteroids have been widely reported to enhance plant resistance to stress, but information on brassinosteroids for the remediation of pesticide-contaminated soils is limited. Under field conditions, brassinosteroids were applied to explore their effects on the residues of tribenuron-methyl, soil enzyme activity, soil microbiol community, and millet yield. After applying brassinosteroids according to the dose of 150 mL hm^−2^, the degradation rate of tribenuron-methyl accelerated. Brassinolide stimulated the activities of catalase and dehydrogenase, while the activities of sucrase and alkaline phosphatase were inhibited. The results of high-throughput sequencing showed that brassinosteroids inhibited the growth of Verrucomicrobia, Ascomycota, and Mortierellomycota and promoted the abundance of cyanobacteria. Additionally, brassinosteroids could also significantly increase the diversity index and change the community structure of soil bacteria and fungi. Further, the predicted function results indicated that brassinosteroids changed some metabolic-related ecological functions of the soil. We also found that brassinolide could increase millet yields by 2.4% and 13.6%. This study provides a theoretical basis for the safe use of tribenuron-methyl in millet fields and a new idea for the treatment of pesticide residues in soil.

## 1. Introduction

Foxtail millet (Setaria italica (L.) P. Beauvois), also known as chestnut, is a gramineous crop that originated in China, and it is rich in nutrients such as protein, vitamins, carotene, and trace elements [1]. Millet is eaten by consumers as porridge or bread. Thanks to its high mullet content, millet also has health benefits, such as lowering blood pressure, promoting fat absorption, and preventing cancer [2]. In China, millet’s potential contribution to national food security has attracted increasing attention from agricultural researchers. At the same time, weeds compete with millet for sunlight, water, and fertilizer, seriously affecting the growth, yield, and quality of millet, resulting in a 20% to 50% reduction in millet production [3]. Compared with traditional manual weeding, chemical weeding has become the main means of weeding in millet fields, due to its advantages of economy and high efficiency.

Tribenuron-methyl (TBM), 2-benzoic acid, belongs to sulfonylurea herbicides and has the characteristics of high efficiency and low toxicity [4]. TBM inhibits acetolactate synthase (ALS) [5], which affects the synthesis of branched-chain amino acids and has a good control effect on broad-leaved weeds, such as Chenopodium and olecranon. Kwiatkowski et al. found that spraying TBM with 18.48 ghm^−2^ and applying nitrogen, phosphorus, potassium, and selenium fertilizer could significantly improve the quality of Jingu 54 [6], which proved the great potential of TBM application in the millet field. At present, 250 TBM products are registered in China, mainly in a single dose (190). TBM has a long persistence in soil, and its non-target impact on the soil environment has attracted some attention [7,8,9,10,11]. Previous studies have shown that TBM seriously damaged the photosynthetic system of Jingu 21 and Yugu 18 [12], caused drug damage to rape [13,14], reduced soil microbial biomass and activity [15], and disturbed the soil microbial community structure. To reduce the toxicity of TBM to non-target organisms, it is necessary to find an effective way to accelerate the degradation of the herbicide in the soil environment.

Brassinosteroid (BR) is a high-efficiency, broad-spectrum sterol plant hormone, considered the sixth largest plant growth regulator after auxin, gibberellin, cytokinin, abscisic acid, and ethylene [16,17]. It has a substantial regulatory effect on the growth and development of plants. Previous studies have shown that BR can enhance the resistance of microbiomes to abiotic and biological stresses [16,18,19]. Faroza et al. found that brassinosteroids increased the photosynthetic efficiency of copper-stressed tomato plants and enhanced the resilience of tomato plants to Cu [20]. Ramirez et al. found that BR improved plant response to cold stress by improving freezing tolerance and optimizing cold adaptation pathways [21]. Wu et al. found that BR increased the activity of antioxidant enzymes in soybean seedlings, reduced the accumulation of reactive oxygen species (ROS), and enhanced the ability of soybean seedlings to withstand saline–alkali stress [22]. Zhou et al. found that 24-epidermis lactone (EBR) could promote the activities of glutathione S-transferase (GST), peroxidase (POD), and glutathione reductase (GR), thus accelerating the metabolisms of chlorpyrifos, cypermethrin, chlorothalonil, and carbendazim, and finally reduce their residue levels in cucumber [23]. However, whether BR affects the bioavailable concentration of TBM in soil and improves the resistance of plants and microorganisms to pesticide stress remains to be studied.

The degradation of TBM in soil depends on microbial metabolism and extracellular catalysis [24]. Inevitably, rational remediation techniques will depend on a thorough dissection of the microorganisms and enzyme activities involved. In this study, based on ultra-high performance liquid chromatography–mass spectrometry (UHPLC-MS/MS) and high-throughput sequencing, BR was used as a remediation material for soil contaminated by TBM to clarify: (1) the dissipation of TBM in soil; (2) the effects of BR on soil microorganisms and enzyme activities; (3) the effect of BR on the degradation of TBM in soil; and (4) the effect of BR on millet yield. In short, as shown in Figure S1, this study aimed to provide a more empirical basis for the repairing of herbicides such as TBM through the application of BR.

## 2. Materials and Methods

### 2.1. Reagents

TBM standard, purity ≥ 99%, was purchased from Beijing Qincheng Yixin Technology Co., Ltd., Beijing, China. The 10% TBM wettable powder was provided by Shengbang Luye Chemical Co., Ltd., Shandong, China; 0.01% 24-epiBR was purchased from Hebei Lansheng Biotechnology Co., Ltd., Hebei, China; and Jingu 21 was provided by the Institute of Economic Crops, Shanxi Academy of Agricultural Sciences, Shanxi, China. Analytical-grade acetonitrile was from Tedia Company, Inc. (Fairfield, OH, USA). LC-grade acetonitrile was purchased from Tedia Corporation (Fairfield, OH, USA). LC-grade formic acid was purchased from Fisher Regent (Beijing, China). LC-grade methanol was from Merck (Darmstadt, Germany). Analytical-grade NaCl and anhydrous MgSO_4_ were from Sinopharm Chemical Reagent Co., Ltd. (Beijing, China). Syringe filters (0.22 µm) were provided by Shimadzu (China). The water used in the experiment was purchased from Guangzhou Watsons Food & Beverage Co., Ltd. (Guangzhou, China).

### 2.2. Field Trial and Soil Sample Collection

A field trial of good agricultural practices was conducted in Taigu District, Jinzhong City, Shanxi Province from May to October 2020. The millet was drilled on 17 May 2020, with a seeding density of 450,000 plants hm^−2^ and a row spacing of 40 cm. Three treatments were set up in the experiment. Each treatment was about 20 m^2^ and was repeated three times: A0, TBM (15.00 g.a.i hm^−2^) without BR; A1, TBM (15.00 g.a.i hm^−2^) with high-dose BR (0.0225 g.a.i hm^−2^); and A2, TBM (15.00 g.a.i hm^−2^) with low-dose BR (0.015 g.a.i hm^−2^). BR and benzenesulfuron were sprayed at the fifth leaf stage of the millet. The topsoils (0~20 cm) collected on the 0th, 1st, 3rd, 7th, 14th, 35th, and 63rd day were put into the refrigerator at −80 °C for TBM residue detection. In addition, the soils collected on the 7th and 60th days were used for high-throughput sequencing and soil enzyme activity determination. All soil collections were removed from small stones and plant roots, passed through a 10-mesh sieve, and stored in a −80 °C refrigerator. During the harvest period, 10 grain panicles were randomly collected from each plot, and the panicle length, panicle width, panicle weight, and thousand-grain weight were measured after detachment and sun-dried to calculate the yield.

### 2.3. TBM Residue Analysis

Tribesulfuron-methyl in soil was extracted by QuEChERS (Quick, Easy, Cheap, Effective, Rugged, Safe) and detected by ultra-high performance liquid chromatography–mass spectrometry (UHPLC-MS/MS, SCIEXTRIPLEQUAD4500, AB Sciex, Framingham, MA, USA) coupled with an electrospray ionization source [25,26]. The 10 g (±0.01 g) soil samples were weighed in a 50 mL centrifuge tube, and 5 mL of purified water and 10 mL of analytical-grade acetonitrile were added; then, it was oscillated violently for about 10 min. Then, 2 g of anhydrous magnesium sulfate and 2 g of sodium chloride were added to vibrate for 5 min violently. After that, it was centrifuged again at 4200 r min^−1^. The above-mentioned supernatant 1.5 mL was quantitatively absorbed into the plastic centrifugal purification tube containing 50 mg of C18 and 3.75 mg of graphitized carbon black (GCB) purification materials and was mixed by the vortex for 1 min. Finally, it was centrifuged at 4200 r min^−1^ for 5 min, and the supernatant was absorbed through the microporous membrane to be determined. TBM was separated by the ACQUITYBEHC18 column (2.1 × 50 mm, 1.7 µm). The mobile phase consisted of 0.1% formic acid aqueous solution (solvent A) and acetonitrile (solvent B). The flow rate was 0.3 mL min^−1^. The binary solvent gradients were as follows: 0–0.5 min at 10% B, 0.5–2 min at 10% to 90% B, 2–3.5 min at 90% to 10% B, 3.5–3.8 min at 90% to 10% B, and then equilibrium at initial conditions for 1.2 min. (The total run time was 5 min.) The column temperature was set at 35 °C, and the volume of the automatic sampler was 1 µL. Retention time (R_t_) and mass spectrometric parameters are listed in Table 1. Multiple reactive ion monitoring (MRM) and positive ion ionization (ESI+) modes were used, with qualitative ion pairs being 396.1 > 155.0 and quantitative ion pairs being 396.1 > 181.0. The ion source temperature was 120 °C, the desolvation temperature was 350 °C, the capillary voltage was 3.0 kV, and the collision gas was argon.

The dissipation curves and half-life (t_1/2_) of TBM in soil were simulated with the first-order kinetic equation:

C_t_ = C_0_e^−kt^ (1)

t_1/2_ = ln2/k (2)

where C_0_ refers to the original deposition (mg kg^−1^). C_t_ represents the residual concentration on day t (mg kg^−1^), and k is the dissipation rate constant.

### 2.4. Detection of Soil Enzyme Activity

Within 20 days after the soil sample was collected, the enzyme activity of the soil passing through a 10-mesh sieve was determined. This study assessed five key soil enzyme activities: catalase, dehydrogenase, sucrase, alkaline phosphatase, and protease. The activities of these enzymes were determined according to the reported methods [27,28]. The catalase activity was measured using potassium permanganate titration; the dehydrogenase activity was measured using the TTC method; the sucrase activity was measured using 3,5-dinitro salicylic acid colorimetry; the phenyl phosphate method was used; and protease activity was determined using the Folin colorimetric method.

### 2.5. Total DNA Extraction and High-Throughput Sequencing

The instructions were followed to extract the total DNA from the soil using E.Z.N.A. ^®^Soil DNA (Omega Bio-Tek, Norcross, GA, USA). The concentration and quality of DNA were determined by agarose gel electrophoresis (1% agarose) and the NanoDrop spectrophotometer (NanoDrop Technologies Inc., Wilmington, NC, USA). The DNA was stored in the −80℃ refrigerator until high-throughput sequencing.

The 16 s rRNA gene of the bacterial V3–V4 region was amplified by standard primers 338F (5′-ACTCCTACGGGAGGCAGAG-3′) and 806R (5′-GGACTACHVGGGTWTCTAAT-3). Additionally, ITS1F (5′-CTTGGTCATTTAGAGGAAGTAA-3′) and ITS2R (5′-GCTGCGTTCTTCATCGATGC-3′) amplified the ITS gene of the fungus. The purified amplification products were sent to Illumina MiSeq PE300 × 2 sequencer for paired-end sequencing. DNA fragment paired-end readings were quality-filtered by fastp version 0.19.6 and merged by flash version 1.2.7 with the following rules: (1) the 300 bp reads were truncated at any site receiving an average quality score of <20 over a 50 bp sliding window, the truncated reads shorter than 50 bp were discarded, and reads containing ambiguous characters were also discarded; (2) only overlapping sequences longer than 10 bp were assembled according to their overlapped sequences. The maximum mismatch ratio of the overlap region was 0.2. Reads that could not be assembled were discarded; (3) samples were distinguished according to the barcode and primers, and the sequence direction was adjusted. Exact barcode matching was performed, with a 2-nucleotide mismatch in primer matching. Then, the QIIME2 feature-classifier plug-in was used to compare the representative sequence of ASV to the GREENGENES database for species annotation. The obtained sequence was leveled with each sample’s minimum number of sequences to obtain the original ASV table. QIIME2 core-diversity plug-in calculated the Alpha diversity index.

### 2.6. Statistical Analysis and Visualization

The residual concentrations of TBM in soil at different times were fitted by the Origin software (version 2017), and the determination coefficient (R^2^) of the equation was obtained. Statistical comparisons of the soil enzyme activity, soil microbial α-diversity index (including Shannon and Chao1 index), and bacterial metabolic pathway abundance were analyzed using one-way analysis of variance (ANOVA), with a post hoc Duncan test using SPSS software (version 25.0), depending on the variance homogeneity test among treatments. Beta diversity index was expressed by the NMDS (non-metric multidimensional scaling) diagram, and R (version 4.3.0) was used to draw the community composition map of each evolutionary classification level. Additionally, the Spearman correlations of TBM residue concentrations, enzyme activities, and microbial diversities were analyzed using R (version 4.3.0).

## 3. Results and Discussion

### 3.1. Residues of TBM in Soil

**Figure 1:**
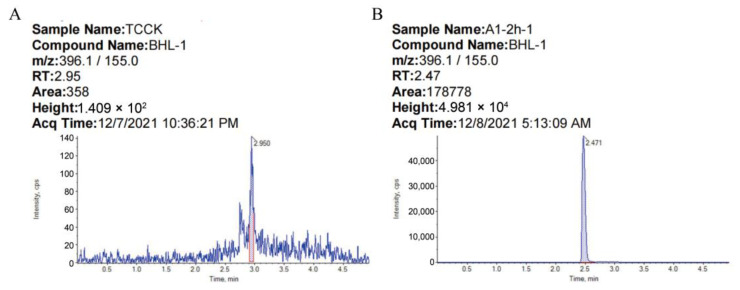
Chromatogram of TBM: (**A**) blank soil; (**B**) soil with 0.01 mg kg^−1^ TBM.

Sulfonylurea herbicides have strong polarity and high water solubility, which can easily pollute groundwater and pose a threat to crops, aquatic organisms, and soil microorganisms and may eventually threaten the health of consumers through the food chain. It is of great significance to establish a simple and economical detection method for the detection of TBM residues to reduce its risk. The QuEChERS pretreatment method was developed in 2003 and has been widely used in the detection of pesticide and veterinary drug residues in food and environmental samples [29,30]. Dong et al. established QuEChERS and rapid-resolution liquid chromatography–tandem mass spectrometry (RRLC-MS/MS) for the determination of TBM in wheat plants, grains, and soil [25]. Using this method, we used acetonitrile as the extraction solvent, sodium chloride and magnesium sulfate for the salting-out process, C18 and GCB as purification materials, and UHPLC-MS/MS to determine TBM in soil. The chromatogram of TBM in soil is shown in Figure 1.

The dissipation of pesticides in soil is usually fitted with first-order kinetic curves [31,32], with the R^2^ ranging from 0.4490 to 0.5323. Compared with A0, the residues of TBM in A1 decreased significantly. The original deposition amounts of TBM in A0, A1, and A2 were 0.012, 0.013, and 0.012 mg kg^−1^, respectively. On the 35th day after application, the residues of TBM in A1 (0.0022 mg kg^−1^) and A2 (0.0027 mg kg^−1^) were significantly lower than those in A0 (0.0030 mg kg^−1^). As shown in Table 2, the half-life of TBM was in the range of 17.8 to 21 days. The half-life in soil reported by Dong et al. was between 1.27 and 10.83 days [25], which was lower than that obtained by us. This might be related to environmental conditions and the application amount of TBM. Compared with A0, the half-lives of TBM in A1 and A2 treatment were shortened by 3.2 days and 0.6 days, respectively, indicating that BR treatment accelerated the degradation of TBM in soil. It was previously reported that the effects of pesticides on soil microorganisms and soil ecological functions were mostly negative. The degradation of TBM in soil included co-metabolism, hydrolysis, and microbial degradation, in which soil microbial communities and enzyme activities were crucial.

### 3.2. Effect of BR on Soil Enzyme Activities with TBM

**Figure 2:**
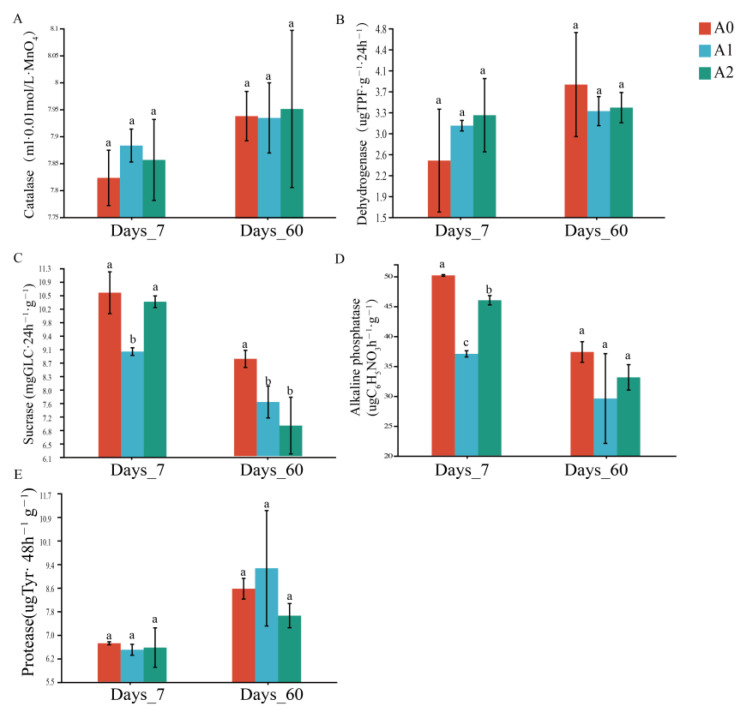
Soil enzyme activities in different treatments. Different letters indicate that there were significant differences between treatments at the same time (*p* < 0.05). (**A**) Catalase; (**B**) dehydrogenase; (**C**) sucrase; (**D**) alkaline phosphatase; (**E**) protease.

Soil enzymes such as catalase, dehydrogenase, sucrase, alkaline phosphatase, and protease play essential roles in material cycling [33]. Soil enzyme activity is affected by soil types, microorganisms, properties, and exogenous stresses. Previous studies have shown that soil enzyme activity is sensitive to environmental stress and can be used to indicate soil environmental pollution. We selected them to determine the changes in soil metabolic capacity under different treatments, as shown in Figure 2. Phytohormones can regulate plant growth at low concentrations and play an important role in the process of plant response to abiotic stress signal transduction. On the 7th day, although it did not reach a significant level (*p* > 0.05), BR stimulated the activities of catalase and dehydrogenase. Similar to our findings, Mazorra et al. found that BR promoted the activity of antioxidant enzymes, such as catalase, in tomatoes [34]. However, the sucrase and alkaline phosphatase activities were significantly decreased (*p* < 0.05) after BR treatment (A1, A2), compared with A0. Sucrase plays an essential role in the soil carbon cycle, and phosphatase activity reflects soil organophosphorus mineralization potential [35]. Unlike our results, Zhou et al. reported that the mixed treatment of BR, gibberellic acid, and microbial agents significantly increased the activities of catalase and sucrase [36]. The level of enzyme activity, which was not only affected by BR, was related to the soil environment and soil microorganisms. Hence, it needs to be further studied with soil microbial composition and community structure.

### 3.3. Effect of BR on Soil Microbiome Community with TBM

**Figure 3:**
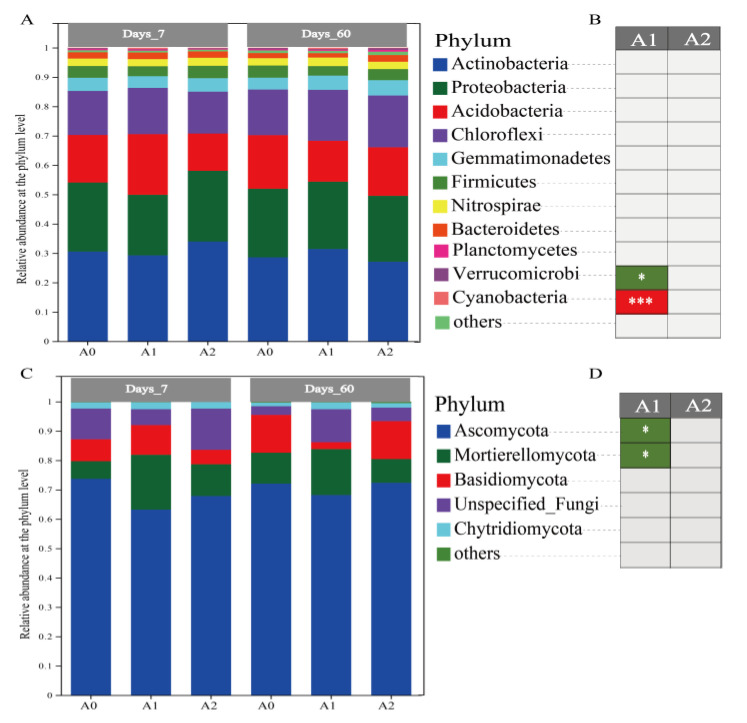
The relative abundances and significance differences of the species on the phylum level. Relative abundances of soil bacteria (**A**) and fungi (**C**) at the phylum level. Compared with A0The species differences of bacteria (**B**) and fungi (**D**) at the phylum level of the BR treatment compared with A0. Green represents an abundance decrease, and red represents an increase. * Represents *p* < 0.05; *** represents *p* < 0.001.

Soil microorganism is an essential part of the farmland soil ecosystem, which is affected by soil properties, plants, and pollutants. Previous reports have shown that pesticides significantly affect soil microbial community structure and ecological function, cause potential harm to the environment, and even interfere with the natural environmental balance [37]. Soil microbes have become the indicators of soil health. In this study, we investigated changes in soil microbial composition and community structure under different treatments to determine whether BR restored soil microbial function under TBM stress. This study used 16SrDNA and ITS high-throughput sequencing technologies to study the structure of soil microbial (soil bacteria and fungi) communities. Totals of 939,801 and 1,068,246 sequences were detected for bacteria and fungi, respectively. Bacteria were annotated into 36 phyla, 118 classes, 212 orders, 336 families, and 513 genera, while fungi were annotated into 10 phyla, 25 classes, 58 orders, 119 families, and 171 genera. Figure 3 shows the relative abundance of bacteria and fungi at the phylum level, with only species with an abundance greater than 1% included. Bacteria mainly consisted of Actinobacteria, Proteobacteria, Acidobacteria, Chloroflexi, Gemmatimonadetes, Firmicutes, Nitrospirae, Bacteroidetes, etc. Fungi were primarily composed of Ascomycota, Mortierellomycota, Basidiomycota, Unspecified_Fungi, and Chytridiomycota. Compared with A0, low-dose BR treatment inhibited the growth of Verrucomicrobia, Ascomycota, and Mortierellomycota and promoted the abundance of cyanobacteria. Verrucomicrobia was dominant in soil bacterial communities, with high diversity and extensive metabolic capacity [38,39]. Its decrease indicated the weakening of soil cellulose metabolism after BR treatment [40].

**Figure 4:**
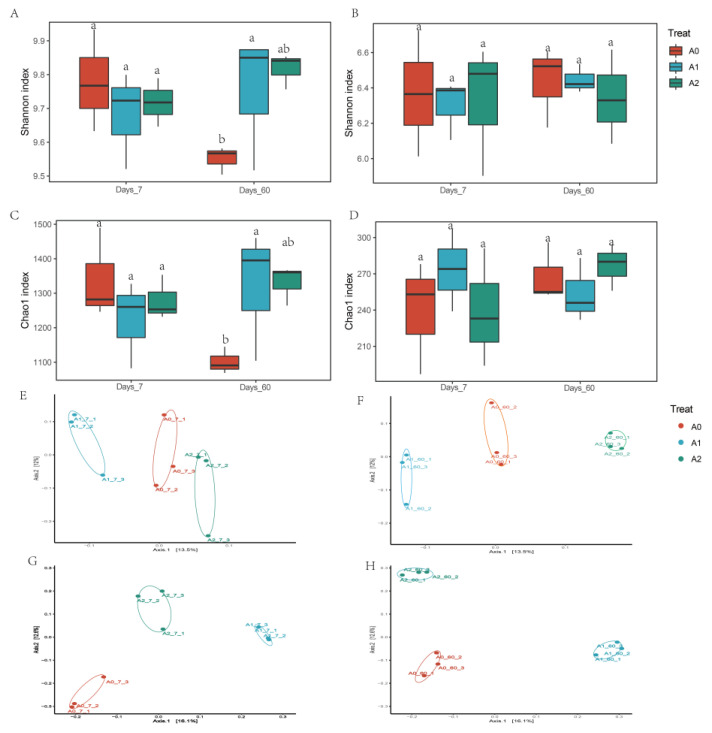
Diversity index of the microbiome. (**A**) Shannon index of bacteria; (**B**) Shannon index of fungi; (**C**) Chao1 index of bacteria; (**D**) Chao1 index of fungi; (**E**) NMDS of bacteria in 7 d; (**F**) NMDS of bacteria in 60 d; (**G**) NMDS of fungi in 7 d; (**H**) NMDS of fungi in 60 d. Different letters indicate that there were significant differences between treatments at the same time (*p* < 0.05).

**Figure 5:**
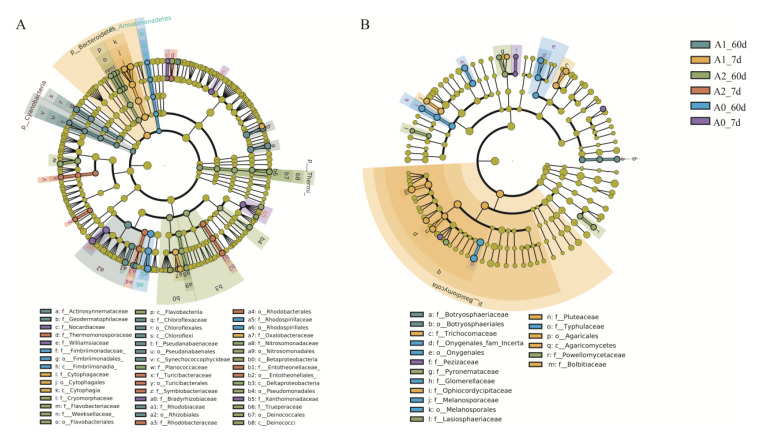
Linear discriminant analysis cladogram diagrams of bacteria (**A**) and fungi (**B**) at different taxonomic levels (LDA > 2).

The α-diversity Chao1 index indicates species richness in the sample, and the Shannon index suggests species diversity. NMDS explores similarities or differences in community composition among different groups of samples through species diversity. The higher the similarity of the bacterial community composition, the closer the distance in the NMDS. As shown in Figure 4, BR significantly increased the Shannon index and Chao1 index of bacteria, indicating that BR promoted the richness and diversity of soil bacterial species. However, the effects of BR on the fungal Shannon index and Chao1 index were insignificant. On the other hand, beta diversity (NMDS) results showed that the BR treatment was separated from the A0 coordinates, indicating that BR significantly changed the community structures of bacteria and fungi. LefSe analysis is an analytical tool to discover and interpret high-dimensional biomarkers [41]. It can be used to compare two or more groups to find statistically different biomarkers between groups. LefSe analysis showed (Figure 5) that there were 45 indicator microorganisms in bacterial treatment and 24 indicator microorganisms in fungi (*p* < 0.05, LDA > 2). During the 7–60 days, significantly enriched species decreased from 7 to 1 under the A1 treatment and increased from 0 to 9 under the A2 treatment. On the 7th day, A1 enhanced seven significant biomarkers: Mortierellaceae, Mortierellomycota, Mortierellales, Mortierellomycetes, Bolbitiaceae, Pluteaceae, and Eurotiales. On the 60th day, only one biomarker of Sordariomycetes was found in the A1 treatment. On the 60th day, A2 enriched nine biomarkers, including Agaricomycetes, Basidiomycota, Ceratobasidiaceae, Cantharellales, Ajellomycetaceae, Gymnoascaceae, Microascaceae, Microascales, and Powellomycetaceae. Mortierella is highly abundant in soils rich in organic matter, plays a crucial role in transforming soil nutrients, and changes the soil environment by affecting soil aggregate [42]. The accelerated degradation of TBM in BR treatment may be related to the enrichment of Mortierella.

Then, we analyzed the Spearman correlations of TBM residue concentrations, enzyme activities, and microbial diversity. As shown in Figure S2, the residual concentration of TBM was significantly positively correlated with invertase (A) and significantly negatively correlated with protease (B), with R being 0.4992 and −0.6465, respectively. Additionally, the Shannon index of fungi had a certain correlation with dehydrogenase (R = 0.4419), although it did not reach a significant level (*p* = 0.066).

### 3.4. Effect of BR on Soil Function with TBM

**Figure 6:**
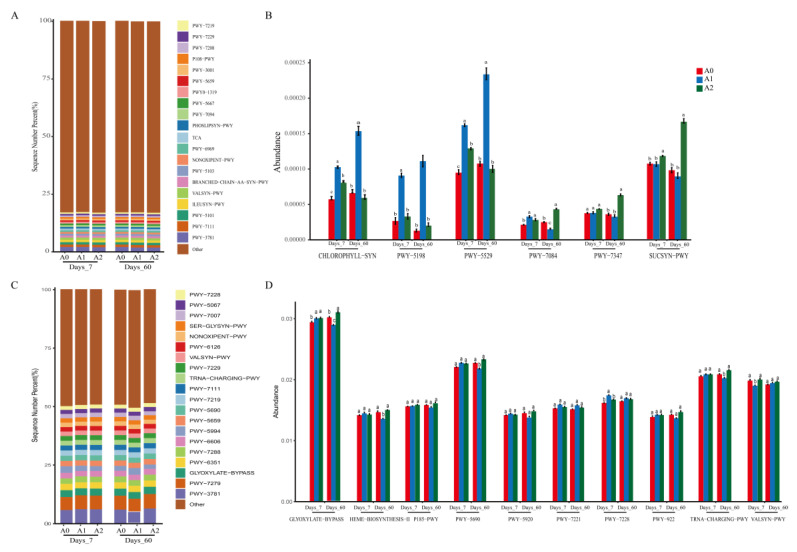
Abundance histogram and difference analysis of the L3 level metabolic pathway based on the KEGG database. Bacterial metabolic pathway abundance histogram (**A**) and difference analysis (**B**); fungal metabolic pathway abundance histogram (**C**) and difference analysis (**D**). Different letters indicate that there were significant differences between treatments at the same time (*p* < 0.05).

Soil microflora plays a vital role in the agro-ecosystem [43]; therefore, further analysis of the impacts of BR and TBM on the soil ecological functions metabolic pathways of bacterial 16s and fungal ITS sequencing data was conducted using the KEGG database (Figure 6). At the L1 level, the proportion of bacterial metabolism was the highest (75.4–75.8%). This was followed by genetic information processing (9.7–9.9%) and cellular processes (5.5–5.7%). Finally, for human diseases, there was organismal systems and environmental information processing. The main pathways of microbial metabolism mapped to the L2 level were amino acid metabolism, carbohydrate metabolism, coenzyme factor, vitamin metabolism, other amino acid metabolisms, lipid metabolism, etc. At the L3 level, 394 signaling pathways were mapped, including valine, leucine, and isoleucine biosynthesis; biosynthesis of terpenoids and steroids; synthesis and degradation of ketone bodies; lipoic acid metabolism; and fatty acid biosynthesis and other metabolic pathways. It is worth noting that there were significant differences in six pathways of MetaCyc, in which A1 treatment increased in the CHLOROPHYLL-SYN (chlorophyllide a biosynthesis I (aerobic, light-dependent), PWY-5198 (factor 420 biosynthesis), and PWY-5529 (super pathway of bacteriochlorophyll a biosynthesis) pathways, and A2 treatment significantly increased in the PWY-7084 (nitrifier denitrification), PWY-7347 (sucrose biosynthesis III), and SUCSYN-PWY (sucrose biosynthesis I (from photosynthesis)) pathways. Fungi sequencing information was mapped to the composition of functional genes, and metabolic pathways were analyzed in the MetaCyc database. The top 20 signaling pathways for fungal abundance included aerobic respiration I (cytochrome c, PWY-3781), aerobic respiration II (cytochromec, yeast, PWY-7279), GLYOXYLATE cycle (GLYOXYLATE BYPASS), triphosphate inositol biosynthesis (PWY-6351), and β-fatty acid oxidation (peroxisome, yeast, PWY-7288). The difference analysis of the MetaCyc pathway showed that GLYOXYLATE BYPASS in the A2 treatment was significantly higher than in the A0 treatment. For A1, pathway I (PWY-7228) for the de novo biosynthesis of guanosine nucleotides was increased considerably, while GLYOXYLATE-BYPASS, tricarboxylic acid cycle II (PWY-5690), tRNA-charging-PWY, and TRNA-CHARGING-PWY increased. In conclusion, brassinosteroids significantly altered some ecological functions related to metabolism.

### 3.5. Effect of BR on Millet Yield

The panicle length, panicle width, panicle weight, thousand-grain weight, and yield are presented in Table 3. BR treatment (A1 and A2) decreased panicle length and panicle weight and increased thousand-grain weight, but it did not affect panicle width. A1 and A2 treatments achieved yield increases of 2.4% and 13.6%, respectively, although they did not reach a significant level, compared with A0. Grain yield was affected by several yield components in varying degrees and was positively correlated with thousand-grain weight. Ning and other studies found that TBM caused Zhangzagu 10 and Jingu 21 to reduce productions by 50.2% and 45.2% [3], respectively. The use of BR could increase the yield, thus reducing the yield loss caused by TBM.

## 4. Conclusions

Sulfonylurea herbicides persist in the soil, which not only cause drug damage to the next crop, they also harm the soil environment. This study used BR to repair millet soil contaminated by TBM. Compared with the control, BR accelerated the degradation of TBM in soil, and the half-life decreased by 3.2 days. In addition, BR also affected soil microbial enzyme activity, bacterial community structure, and soil ecological function under TBM pollution. We also found that BR increased millet yields by 2.4% (A1) and 13.6% (A2). Although our studies show that BR has great potential in the remediation of pesticide-contaminated soils, further studies are needed to evaluate the mechanism and efficiency of BR remediation under field conditions.

## Figures and Tables

**Table 1:** Chemical structure and mass spectrometric parameters of TBM.

Structural Formula	Retention Time (min)	Parent Ion (*m*/*z*)	Product Ion (*m*/*z*)	Collision Energy (eV)
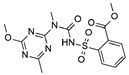	2.5	396.1	155.0	21
			181.0	27

**Table 2:** The first-order kinetic equation and half-life of TBM dissipation in different treated soils.

Treat	First-Order Kinetic Equation	R^2^	Half-Life (Days)
A0	C_t_ = 0.0125e^−0.033x^	0.4490	21.0
A1	C_t_ = 0.0132e^−0.039x^	0.5323	17.8
A2	C_t_ = 0.0121e^−0.034x^	0.4499	20.4

**Table 3:** Effects of treatment on panicle length, panicle width, panicle weight, 1000-grain weight, and yield of millet.

Treatments	Panicle Length (cm)	Panicle Width (mm)	Panicle Weigth (g)	Thousand-Grain Weight (g)	Yield (kg hm^−2^)
A0	25.06 ± 1.65 a	27.64 ± 4.76 a	24.2 ± 6.37 a	3.17 ± 0.02 b	4447 ± 27.3 a
A1	22.12 ± 2.75 b	24.08 ± 4 a	22.17 ± 6.26 ab	3.17 ± 0.04 b	4552 ± 28.0 a
A2	22.03 ± 2.39 b	26.3 ± 4.28 a	16.39 ± 4.88 b	3.24 ± 0.02 a	5054 ± 25.4 a

Different letters represent significant differences between treatments (*p* < 0.05).

## Data Availability

Data is contained within the article or supplementary material.

## Supplementary Materials

The following supporting information can be downloaded at: https://www.mdpi.com/article/10.3390/microorganisms11071829/s1, Figure S1: BR promotes TBM degradation by altering enzyme activity and microbial composition, and ultimately affects millet yield; Figure S2: Spearman correlation analysis. Correlation between residual concentration of TBM and sucrase (A) and protease (B); Correlation between shannon index and dehydrogenase (C).

## References

[1] Saleh A.S.M., Zhang Q., Chen J., Shen Q. (2013). Millet Grains: Nutritional Quality, Processing, and Potential Health Benefits. Compr. Rev. Food Sci. Food Saf..

[2] Devi P.B., Vijayabharathi R., Sathyabama S., Malleshi N.G., Priyadarisini V.B. (2014). Health benefits of finger millet (*Eleusine coracana* L.) polyphenols and dietary fiber: A review. J. Food Sci. Technol.-Mysore.

[3] Ning N., Yuan X.Y., Dong S.Q., Wen Y.Y., Gao Z.P., Guo M.J., Guo P.Y. (2015). Grain Yield and Quality of Foxtail Millet (*Setaria italica* L.) in Response to Tribenuron-Methyl. PLoS ONE.

[4] McCourt J.A., Pang S.S., Guddat L.W., Duggleby R.G. (2005). Elucidating the specificity of binding of sulfonylurea herbicides to acetohydroxyacid synthase. Biochemistry.

[5] Deng W., Di Y., Cai J., Chen Y., Yuan S. (2019). Target-Site Resistance Mechanisms to Tribenuron-methyl and Cross-resistance Patterns to ALS-inhibiting Herbicides of Catchweed Bedstraw (*Galium aparine*) with Different ALS Mutations. Weed Sci..

[6] Yang Y.J., Guo M.J., Pang Y.F., Shao Q.L., Wu Y.Z., Zhao H.M., Ji A.Q., Ma J.H., Song X.E., Sun C.Q. (2021). Foxtail millet (setaria italica (L.) P. Beauvois) quality response to fertilizer level, herbicide and selenium. Appl. Ecol. Environ. Res..

[7] Duman F., Urey E., Temizgul R., Bozok F. (2010). Biological responses of a non-target aquatic plant (*Nasturtium officinale*) to the herbicide, tribenuron-methyl. Weed Biol. Manag..

[8] Hu M., Zhang H., Kong L., Ma J., Wang T., Lu X., Guo Y., Zhang J., Guan R., Chu P. (2023). Comparative proteomic and physiological analyses reveal tribenuron-methyl phytotoxicity and nontarget-site resistance mechanisms in *Brassica napus*. Plant Cell Environ..

[9] Kayhan F.E., Duruel H.E.E., Kizilkaya S., Dine S.K., Kaymak G., Akbulut C., Ertug N.D.Y. (2020). Toxic effects of herbicide tribenuron-methyl on liver tissue of zebrafish (Danio Rerio). Fresenius Environ. Bull..

[10] Kokojka F., Bacu A., Shahini S., Medha G. (2023). Tribenuron-methyl treatment affects glutathione metabolization and other physiological processes in bread wheat. Int. J. Pest Manag..

[11] Liu W., Bai S., Zhao N., Jia S., Li W., Zhang L., Wang J. (2018). Non-target site-based resistance to tribenuron-methyl and essential involved genes in *Myosoton aquaticum* (L.). BMC Plant Biol..

[12] Chang Z., Wang Y., Zhao X., Wang Y., Ma K., Yuan X., Dong S. (2021). Effects of ‘tribesulfuron-methyl’ on photosynthetic characteristics of different foxtail millet varieties. J. China Agric. Univ..

[13] Lian J.-L., Ren L.-S., Zhang C., Yu C.-Y., Huang Z., Xu A.-X., Dong J.-G. (2019). How exposure to ALS-inhibiting gametocide tribenuron-methyl induces male sterility in rapeseed. BMC Plant Biol..

[14] Mehdizadeh M., Alebrahim M.T., Roushani M., Streibig J.C. (2016). Evaluation of four different crops’ sensitivity to sulfosulfuron and tribenuron methyl soil residues. Acta Agric. Scand. Sect. B-Soil Plant Sci..

[15] Rachedi K., Zermane F., Tir R., Ayache F., Duran R., Lauga B., Karama S., Simon M., Boulahrouf A. (2018). Effect of sulfonylurea tribenuron methyl herbicide on soil Actinobacteria growth and characterization of resistant strains. Braz. J. Microbiol..

[16] Clouse S.D., Sasse J.M. (1998). BRASSINOSTEROIDS: Essential Regulators of Plant Growth and Development. Annu. Rev. Plant Physiol. Plant Mol. Biol..

[17] Fujioka S., Yokota T. (2003). Biosynthesis and metabolism of brassinosteroids. Annu. Rev. Plant Biol..

[18] Bajguz A., Hayat S. (2009). Effects of brassinosteroids on the plant responses to environmental stresses. Plant Physiol. Biochem..

[19] Nolan T.M., Vukasinovic N., Liu D., Russinova E., Yin Y. (2020). Brassinosteroids: Multidimensional Regulators of Plant Growth, Development, and Stress Responses. Plant Cell.

[20] Nazir F., Fariduddin Q., Hussain A., Khan T.A. (2021). Brassinosteroid and hydrogen peroxide improve photosynthetic machinery, stomatal movement, root morphology and cell viability and reduce Cu-triggered oxidative burst in tomato. Ecotoxicol. Environ. Saf..

[21] Ramirez V.E., Poppenberger B. (2020). Modes of Brassinosteroid Activity in Cold Stress Tolerance. Front. Plant Sci..

[22] Wu Y., Gao H., Zhang B., Zhang H., Wang Q., Liu X., Luan X., Ma Y. (2017). Effects of 24-Brassinolide on the Fertility, Physiological Characteristics and Cell Ultra-Structure of Soybean Under Saline-Alkali Stress. Sci. Agric. Sin..

[23] Zhou Y., Xia X., Yu G., Wang J., Wu J., Wang M., Yang Y., Shi K., Yu Y., Chen Z. (2015). Brassinosteroids play a critical role in the regulation of pesticide metabolism in crop plants. Sci. Rep..

[24] Burns R.G., DeForest J.L., Marxsen J., Sinsabaugh R.L., Stromberger M.E., Wallenstein M.D., Weintraub M.N., Zoppini A. (2013). Soil enzymes in a changing environment: Current knowledge and future directions. Soil Biol. Biochem..

[25] Dong B.Z., Qian W., Hu J.Y. (2015). Dissipation kinetics and residues of florasulam and tribenuron-methyl in wheat ecosystem. Chemosphere.

[26] Anastassiades M., Lehotay S.J., Stajnbaher D., Schenck F.J. (2003). Fast and easy multiresidue method employing acetonitrile extraction/partitioning and “dispersive solid-phase extraction” for the determination of pesticide residues in produce. J. AOAC Int..

[27] Gianfreda L., Antonietta Rao M., Piotrowska A., Palumbo G., Colombo C. (2005). Soil enzyme activities as affected by anthropogenic alterations: Intensive agricultural practices and organic pollution. Sci. Total Environ..

[28] Perucci P., Casucci C., Dumontet S. (2000). An improved method to evaluate the o-diphenol oxidase activity of soil. Soil Biol. Biochem..

[29] Garrido Frenich A., Romero-Gonzalez R., Luz Gomez-Perez M., Martinez Vidal J.L. (2011). Multi-mycotoxin analysis in eggs using a QuEChERS-based extraction procedure and ultra-high-pressure liquid chromatography coupled to triple quadrupole mass spectrometry. J. Chromatogr. A.

[30] Chen G., Cao P., Liu R. (2011). A multi-residue method for fast determination of pesticides in tea by ultra performance liquid chromatography-electrospray tandem mass spectrometry combined with modified QuEChERS sample preparation procedure. Food Chem..

[31] Cao J., Lv Y., Qi Y., Qin S., Wang X., Li J. (2022). Dissipation, terminal residue and dietary risk assessment of flonicamid in cabbage. Int. J. Environ. Anal. Chem..

[32] Zhao J., Tan Z., Wen Y., Fan S., Liu C. (2020). Dissipation of fluazinam in citrus groves and a risk assessment for its dietary intake. J. Sci. Food Agric..

[33] Neemisha, Sharma S. (2022). Soil enzymes and their role in nutrient cycling. Structure and Functions of Pedosphere.

[34] Mazorra L.M., Nunez M., Hechavarria M., Coll F., Sánchez-Blanco M.J. (2002). Influence of brassinosteroids on antioxidant enzymes activity in tomato under different temperatures. Biol. Plant.

[35] Dick R.P. (1994). Soil enzyme activities as indicators of soil quality. Defin. Soil Qual. A Sustain. Environ..

[36] Zhou X., Shu R., Huang H., Yang S., Long Y., Liu W., Yin X. (2018). Effects of different remediate agents on tobacco plants caused phytotoxicity by quinclorac and its soil microorganisms and enzyme activities. J. South. Agric..

[37] Wang G., Li X., Xi X., Cong W.-F. (2022). Crop diversification reinforces soil microbiome functions and soil health. Plant Soil.

[38] Bergmann G.T., Bates S.T., Eilers K.G., Lauber C.L., Caporaso J.G., Walters W.A., Knight R., Fierer N. (2011). The under-recognized dominance of Verrucomicrobia in soil bacterial communities. Soil Biol. Biochem..

[39] Fuerst J.A., Schmidt T.M. (2019). Phylum Verrucomicrobia. Encyclopedia of Microbiology.

[40] Wang X., Bian Q., Jiang Y., Zhu L., Chen Y., Liang Y., Sun B. (2021). Organic amendments drive shifts in microbial community structure and keystone taxa which increase C mineralization across aggregate size classes. Soil Biol. Biochem..

[41] Chang F., He S., Dang C. (2022). Assisted selection of biomarkers by linear discriminant analysis effect size (LEfSe) in microbiome data. JoVE (J. Vis. Exp.).

[42] Li F., Chen L., Zhao Z.-H., Li Y., Yu H.-Y., Wang Y., Zhang J.-B., Han Y.-L. (2023). The changes of chemical molecular components in soil organic matter are associated with fungus *Mortierella capitata* K. Soil Tillage Res..

[43] Yadav S.K., Soni R., Rajput A.S. (2018). Role of microbes in organic farming for sustainable agro-ecosystem. Microorganisms for Green Revolution: Volume 2: Microbes for Sustainable Agro-Ecosystem.

