# Supplementary material for: Effect of Brassinolide on Soil Microorganisms in Millet Field Polluted by Tribenuron-Methyl
Source: Microorganisms. 2023 Jul 18;11(7):1829. doi: 10.3390/microorganisms11071829 (PMC10384783; doi:10.3390/microorganisms11071829)
Supplement: Supplementary file 1 [file microorganisms-11-01829-s001.zip › microorganisms-2456747-supplementary.pdf]

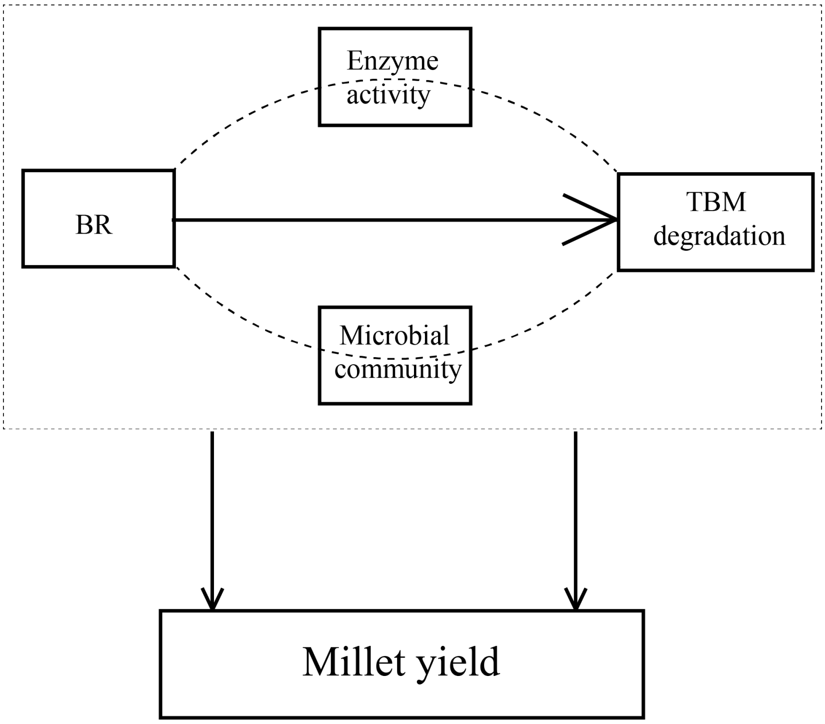

Figure S1. BR promotes TBM degradation by altering enzyme activity and microbial composition, and ultimately affects millet yield

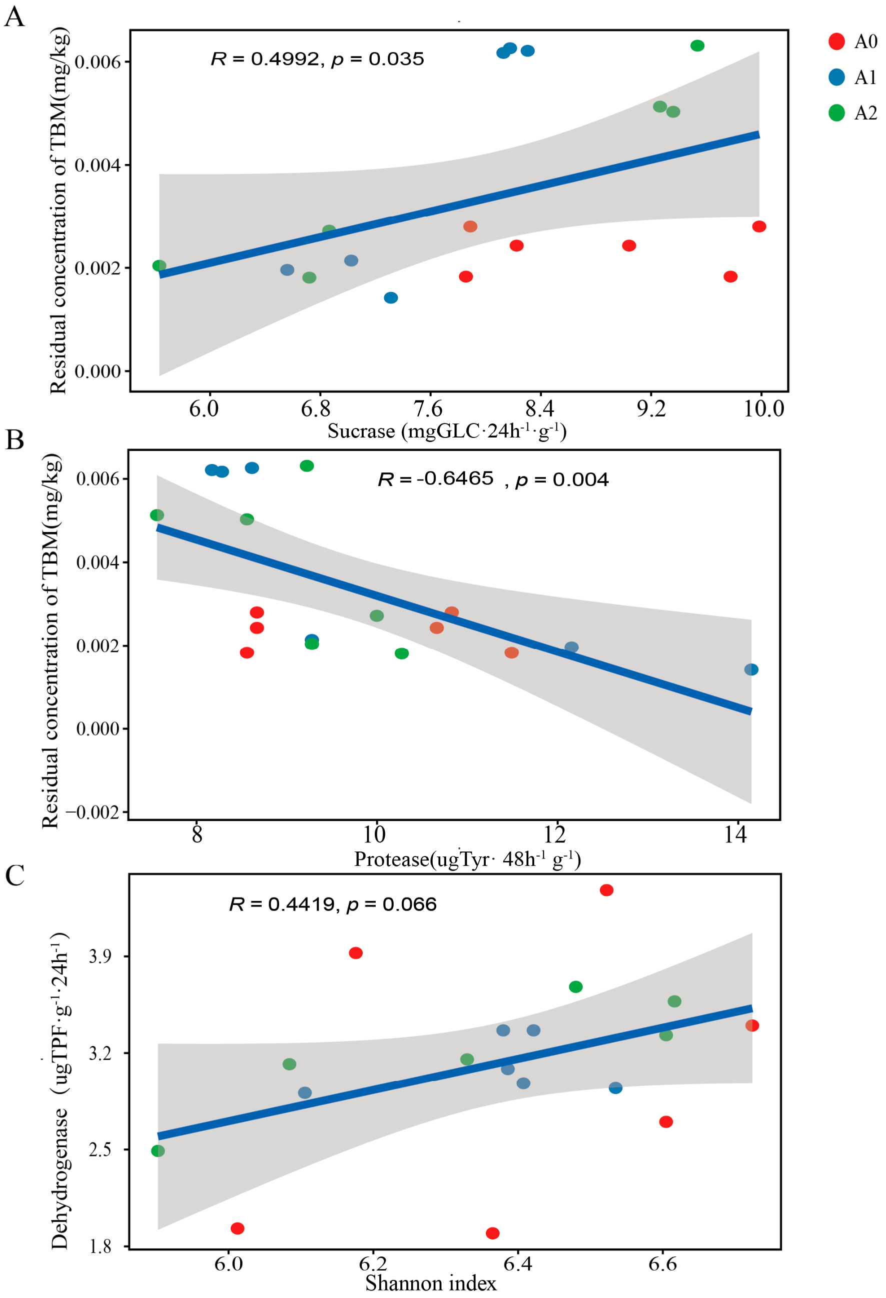

Figure S2. Spearman correlation analysis. Correlation between residual concentration of TBM and sucrase (A) and protease (B); Correlation between shannon index and dehydrogenase (C).
